# 3D evaluation of maxillary morphology in Marfan growing subjects: a controlled clinical study

**DOI:** 10.1186/s40510-019-0264-2

**Published:** 2019-03-18

**Authors:** Giuseppina Laganà, Daniel Palmacci, Giovanni Ruvolo, Paola Cozza, Valeria Paoloni

**Affiliations:** 1 Department of Clinical Sciences and Translational Medicine, University of Rome Tor Vergata, via Montpellier 1, 00133 Rome, Italy; 2 Department of Cardiac Surgery Unit, Centre for Rare Diseases for Marfan Syndrome and Related Disorders, University of Tor Vergata General Hospital, Rome, Italy

**Keywords:** Marfan syndrome, Maxillary morphology, Geometric morphometric analysis, 3d evaluation

## Abstract

**Background:**

Marfan syndrome is a rare autosomal dominant inherited disease of the connective tissue associated with various craniofacial abnormalities. Aim of the present study was to assess the variability of palatal shape in a sample of 31 Marfan patients compared to a control group of no syndromic subjects, in two stages of dentition, by using 3D geometric morphometric analysis.

**Methods:**

Thirty one growing subjects with Marfan syndrome were selected and divided into two subgroups: MG1 with mixed dentition (10 M, 6F, mean age 7+/− 0.7 years), MG2 with permanent dentition (8 M, 7F, mean age 13+/− 0,5 years). Each subgroup was compared to a control group (CG1 mixed dentition, 9 M, 7F, mean age 7.6+/− 0.5 years; CG2 permanent dentition, 9 M, 6F, mean age 12.8+/− 0.7 years) matched on age, sex distribution, stage of dentition and skeletal maturation. Then the two subgroups were compared one to each other. For each patient maxillary dental casts were taken, scanned and digitized. 3D geometric morphometric methods were applied. Procrustes analysis was used and principal component analysis was performed to reveal the main patterns of palatal shape variation.

**Results:**

Both Marfan subgroups showed important reductions in the transversal plane associated with a deep palatal vault when compared to the control groups (MG1 vs CG1 *P* = 0,003; MG2 vs CG2 P = 0,07). Moreover a statistically significant difference between the palatal shape of MG1 and MG2 was found (*P* = 0.017) showing a significant worsening of palatal depth and constriction from mixed to permanent dentition in Marfan subjects.

**Conclusion:**

Marfan subjects showed a specific palatal morphology with maxillary constriction and deeper palatal vault when compared to a control group of healthy subjects. The constriction and the depth of the palatal vault in Marfan patients worsen from mixed dentition to permanent dentition more then in no syndromic subjects.

## Background

Marfan syndrome (MS) is a rare disorder of connective tissue that can affect heart, blood vessels, lungs, eyes, bones, and ligaments. The condition was named after a French pediatrician, Antoine Bernard-Jean Marfan, who first described its occurrence in 1896 in a 5-year-old girl named Gabrielle with “spider’s legs” or dolicostenomely (from the Greek: stenos = narrow, slender; melos = limb); the patient was noted to have disproportionately long and thin arms, legs, fingers, and toes [1]. Mutations in the FBN1 gene, which encodes the matrix protein fibrillin 1, are the predominant causes of classic Marfan syndrome. In fact only 25% of the sporadic cases lead to de novo mutations in zones, which are distant several base pairs from this gene sequence, is documented [2]. Its prevalence has been estimated at 2–3 persons per 10,000, but data are not always confirmed [3]. The diagnosis of Marfan syndrome is based on a combination of the major and minor clinical features described in the 1986 Berlin classification system, which was revised by expert consensus to create the 1996 Ghent classification system [4]. A number of oral manifestations, such as high incidence of caries, tooth root deformity, abnormal pulp chambers with obliteration and high susceptibility to periodontal pathologies, have been reported to Marfan syndrome [5, 6]. Craniofacial abnormalities include dolichocephaly (long face), highly arched palate, maxillary and mandibular retrognathia and macrocephaly. Maxillary constriction and high-arched palate, concomitant with crowding and posterior cross-bite, and skeletal Class II malocclusions are commonly noted [7, 8]. In literature few data are available with regard to three-dimensional morphology of the palatal vault in Marfan syndrome. Cistulli et al. evaluated maxillary characteristics in Marfan patients by linear measurements such as intermolar and intercanine distances. Docimo et al. analyzed their upper jaws by clinical and radiological observation [9].

To our knowledge only one preliminary study [10] evaluated the palatal vault in 3D, but the collected sample was of only just five male patients with Marfan syndrome.

The aim of the present study was to assess the variability of palatal shape in a sample of 31 Marfan patients compared to a control group of no syndromic subjects, in two different stages of dentition, by using 3D geometric morphometric analysis.

## Methods

This project was approved by the Ethical Committee of the University of Rome “Tor Vergata” (Protocol number: 4544/2017) and informed consent was obtained from the patients’ parents.

A sample of 31 subjects (20 males, 11 females) with a clinical diagnosis of Marfan syndrome was recruited from the Centre for Rare Disease, Marfan Clinic of Rome “Tor Vergata” University Hospital and evaluated in the Department of Orthodontics of the same University.

Inclusion criteria were: genetic assessment of MS and Caucasian ancestry. Exclusion criteria were: post pubertal stage (CS5-CS6) [11], previous orthodontic treatment, deciduous dentition.

Then the 31 Marfan subjects were divided in two subgroups according to the stage of dentition and they were compared one to each other. The first subgroup (MG1) was composed of 16 subjects (10 males, 6 females, mean age 7+/− 0.7 years) with mixed dentition. The second one (MG2) was composed of 15 subjects (8 males, 7 females, mean age 13+/− 0,5 years) with permanent dentition.

Moreover a control group of 31 subjects was collected using the following inclusion criteria: no syndromic subjects, Caucasian ancestry, good occlusion, no previous orthodontic treatment, no deciduous stage of dentition, no post pubertal stage of skeletal maturation (CS5, CS6) [11]. The control group in mixed dentition was called CG1 (9 M, 7F, mean age 7.6+/− 0.5 years); CG2 for the one permanent dentition (9 M, 6F, mean age 12.8+/− 0.7 years).

MG1 matched CG1 and MG2 matched CG2 in terms of age, sex distribution, dentition stage and skeletal maturation.

**Fig. 1:**
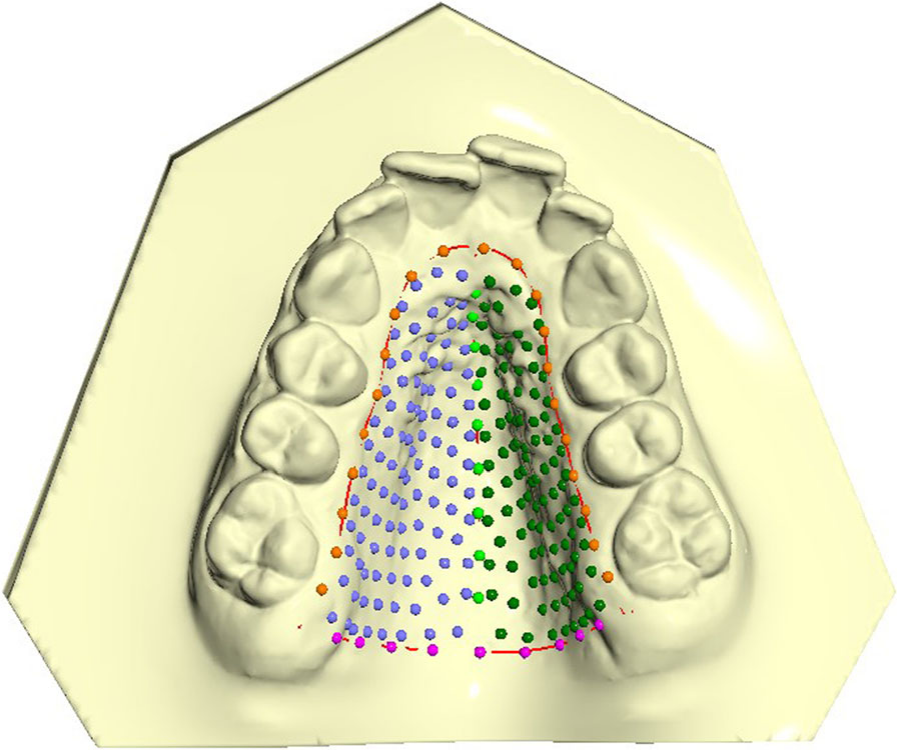
The template of 240 landmarks and semi-landmarks used to study the palatal shape

For each subject dental casts were taken before any treatment. Maxillary study casts of all subjects were scanned using the extraoral scanner OrthoXscan (OrthoXscan; Dentaurum GmbH&co, Ispringen, Germany) with a manufacturer’s reported accuracy of < 20 μm. All models were exported in a Standard Tesselation Language format (.stl digital file). To study the palatal shape, 3D geometric morphometric (GMM) analysis was used [12–14]. A template for data set/collection of homologous landmarks describing a palate was created with Viewbox 4 (dHAL software, Kifissia, Greece). On each digital cast, three curves were drawn and a total of 239 landmarks were digitized [15]. The boundaries of the palate were defined as: the midsagittal suture (9 points), a perimeter curve of the dental arch passing apical to the gingival sulci of each tooth (21 points) and a posterior curve passing from distal of the first permanent molars, perpendicular to the midsagittal line (9 points). The remaining points (semilandmarks) were placed uniformly on the palatal surface within the confines delimited by the three curves [16] (Fig. 1). The averages of all the datasets were calculated and used as a fixed reference (Procrustes average) to allow all semilandmarks to slide and become more homologous from subject to subject in order to minimize the thin-plate spline (TPS) bending energy [17–19]. This procedure was repeated three times. All digitizations of study casts were performed by the same operator (V.P.) and analyzed using the Generalized Procrustes method.

Procrustes analysis was applied and principal component analysis (PCA) was performed to reveal the main patterns of palatal shape variation [19]. Procrustes superimposition was used to evaluate the statistical differences between the groups. More than 10,000 permutations have been reported.

To determine the reliability of the method, 20 casts were re-digitized by the same operator ten days after the first digitization.

The following comparisons were analyzed: MG1 vs MG2, MG1 vs CG1, MG2 vs CG2, CG1 vs CG2.

## Results

Mean random error of the 20 repeated digitizations, expressed as a percentage of total shape variance, was 2.3%. No statistically significance differences between the different genders were found.

When comparing MG1 vs MG2 a statistically significant difference between their palate’s shape was found (10000 permutations; *P* = 0.017).

**Fig. 2:**
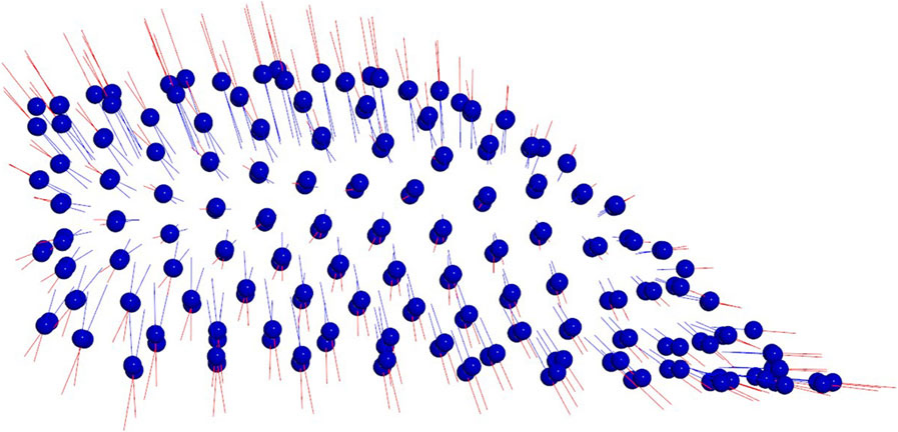
Results of PC1 from a lateral view. Blue dots represent the average of the palatal shape; blue lines show the trend of MG1; red lines show the trend of MG2

**Fig. 3:**
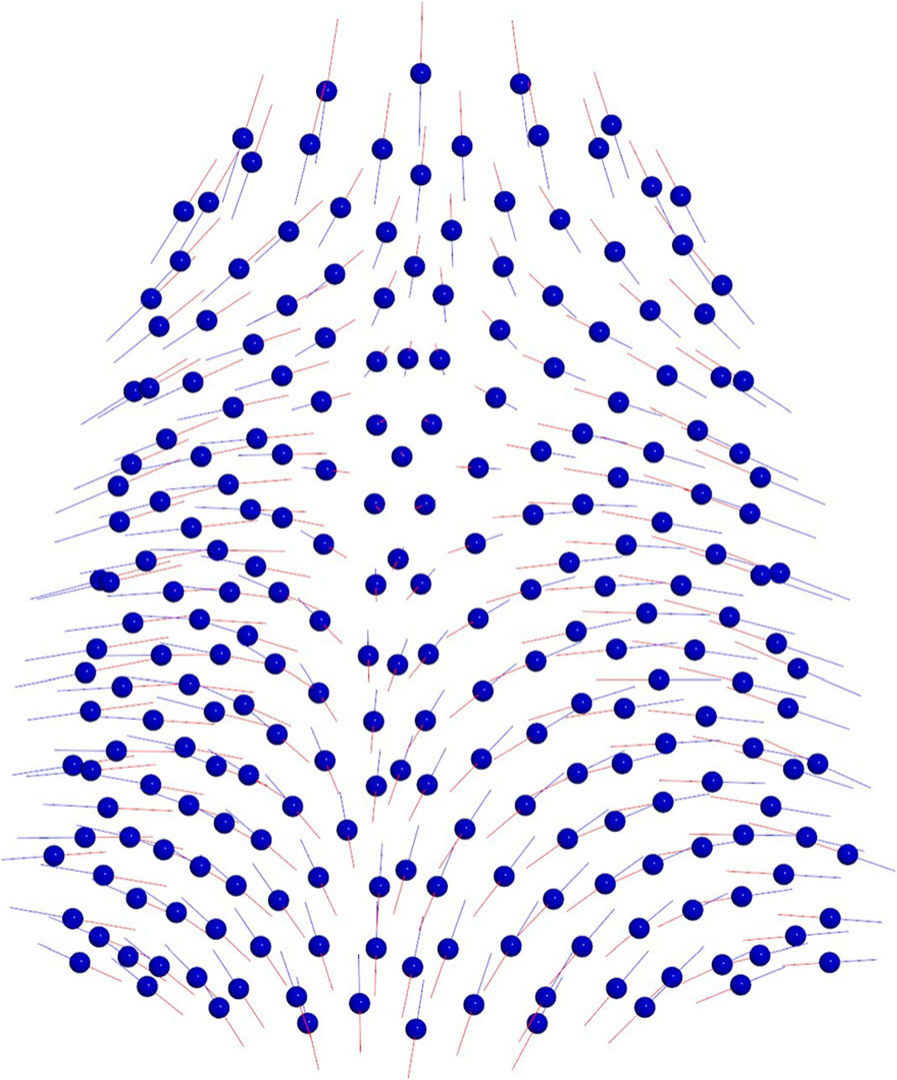
Results of PC1 from a superior view. Blue dots represent the average of the palatal shape; blue lines show the trend of MG1; red lines show the trend of MG2

**Fig. 4:**
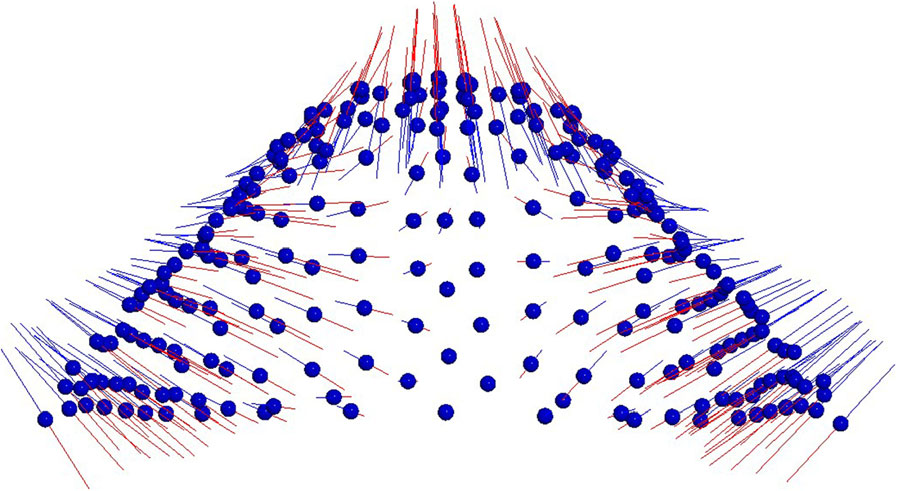
Results of PC1 from a posterior view. Blue dots represent the average of the palatal shape; blue lines show the trend of MG1; red lines show the trend of MG2

**Fig. 5:**
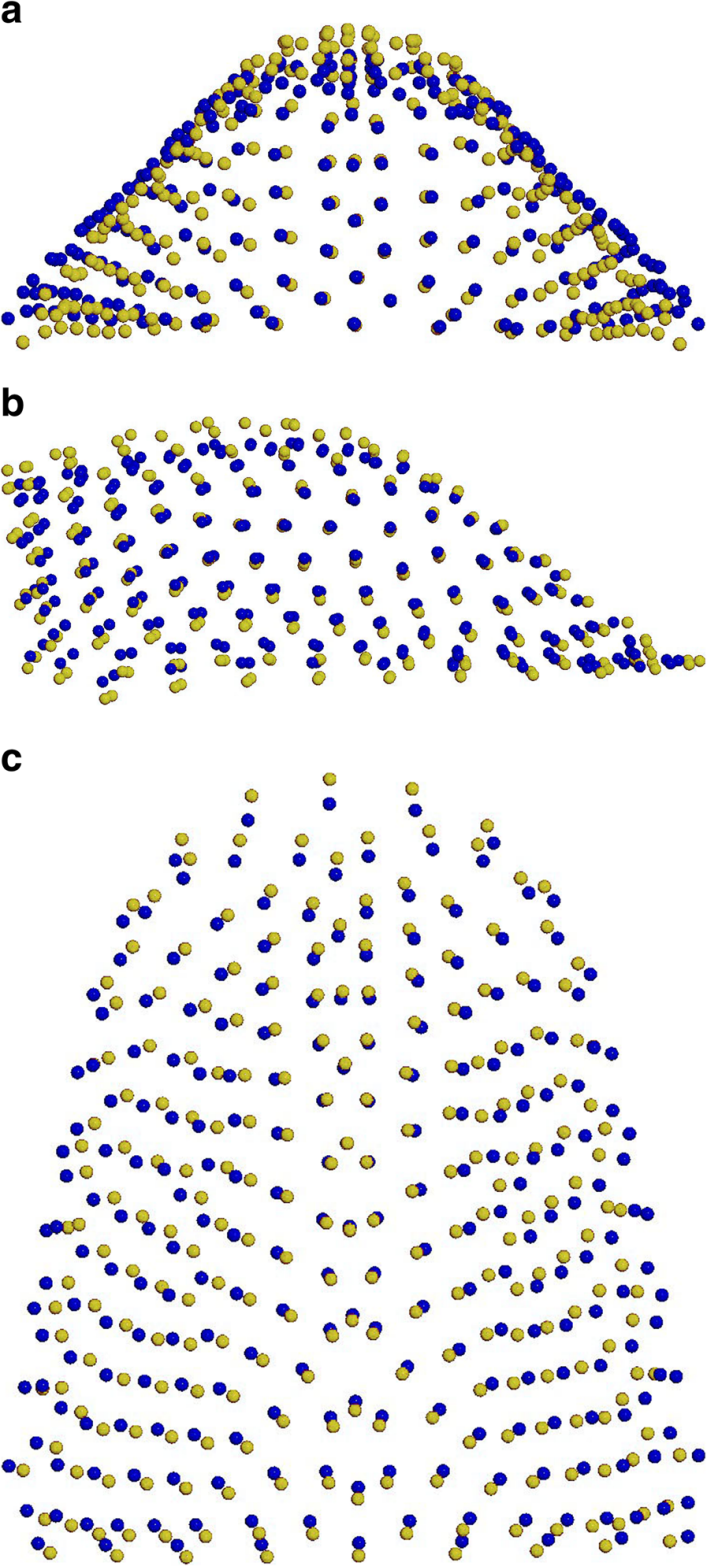
**a**-**c** Comparison between the average palatal shape of MG1 (blue) and MG2 (yellow): **a** posterior view; **b** sagittal view; **c** superior view

The first two principal components (PCs) composed the 64% of total shape variability (PC1 = 40,7%, PC2 = 23,5%). The variability described by the first principal component (PC1) was morphologically the most significant and defines the 40,7% of total shape variability. PC1 showed shape variations in two dimensions of space. More specifically on a lateral view, PC1 shows a higher palate’s conformation in MG2 (Fig. 2). On a superior view (Fig. 3), the palate of MG2 appears constricted in the anterior region of the maxilla and it presents a marked reduction gradient to the posterior region of the palate. Observing the palate from the posterior view (Fig. 4) it is possible to confirm the severe transverse constriction in Marfan group with permanent dentition with higher and narrowed palatal vault when compared with Marfan group with mixed dentition. Figure 5a-c shows the comparison between MG1 (blue points) and MG2 (red points) palatal shape on lateral, superior and posterior views. Significant changes were particularly related to a deformation located on lateral and posterior regions. In particular, MG2 showed a narrowed and higher palate compared to MG1.

**Fig. 6:**
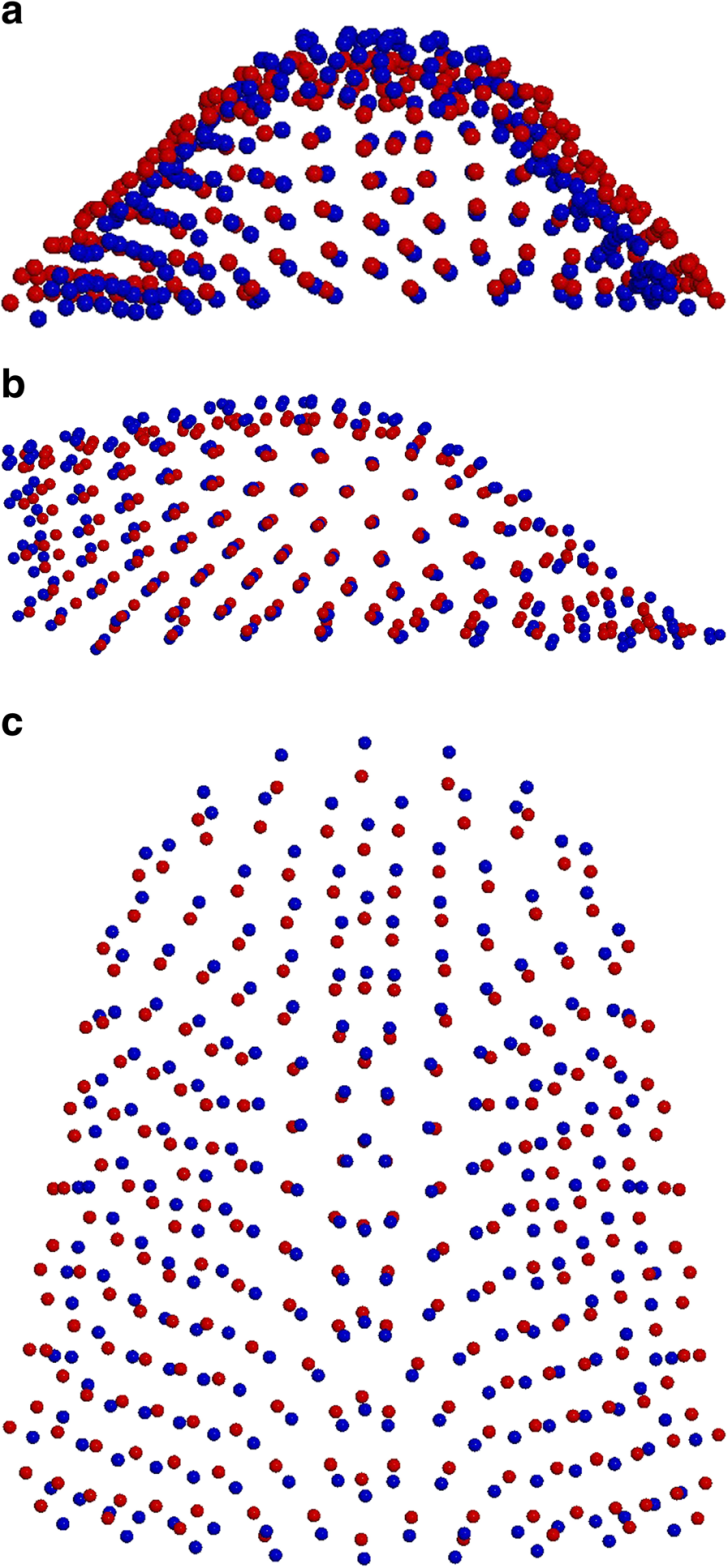
**a**-**c** Comparison between MG1 (blue) and CG1 (red): **a** posterior view; **b** sagittal view; **c** superior view

**Fig. 7:**
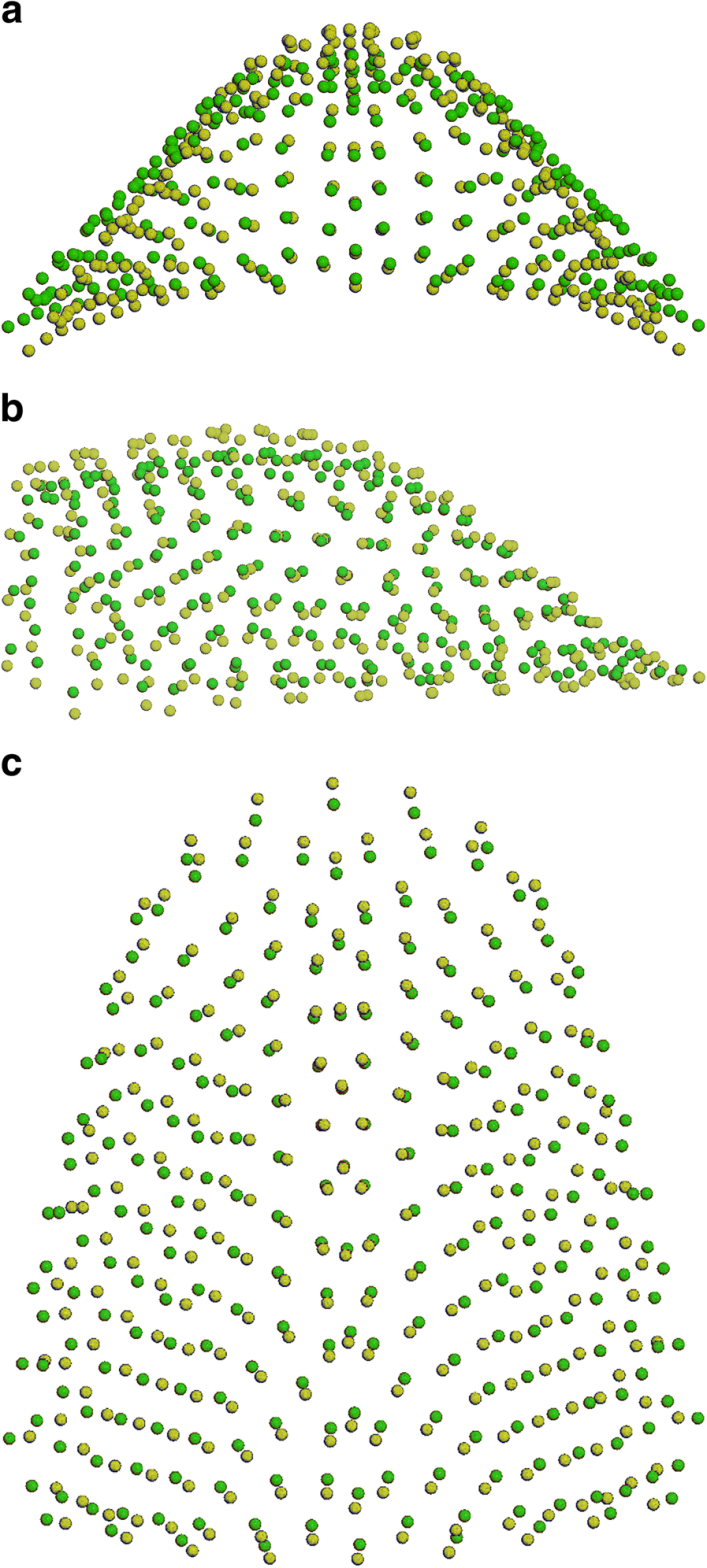
**a**-**c** Comparison between MG2 (yellow) and CG2 (green): **a** posterior view; **b** sagittal view; **c** superior view

**Fig. 8:**
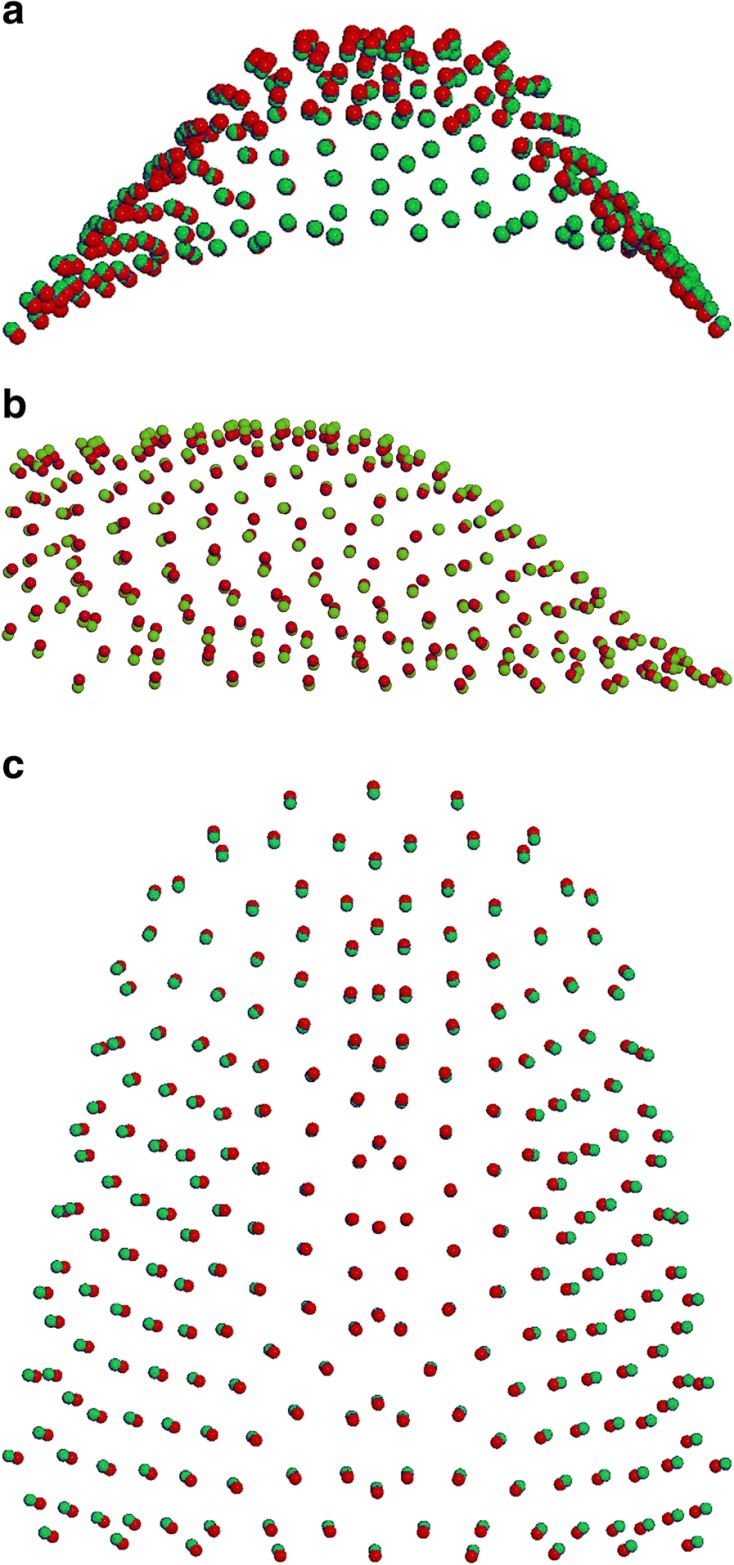
**a**-**c** Comparison between CG1 (red) and CG2 (green): **a** posterior view; **b** sagittal view; **c** superior view

**Fig. 9:**
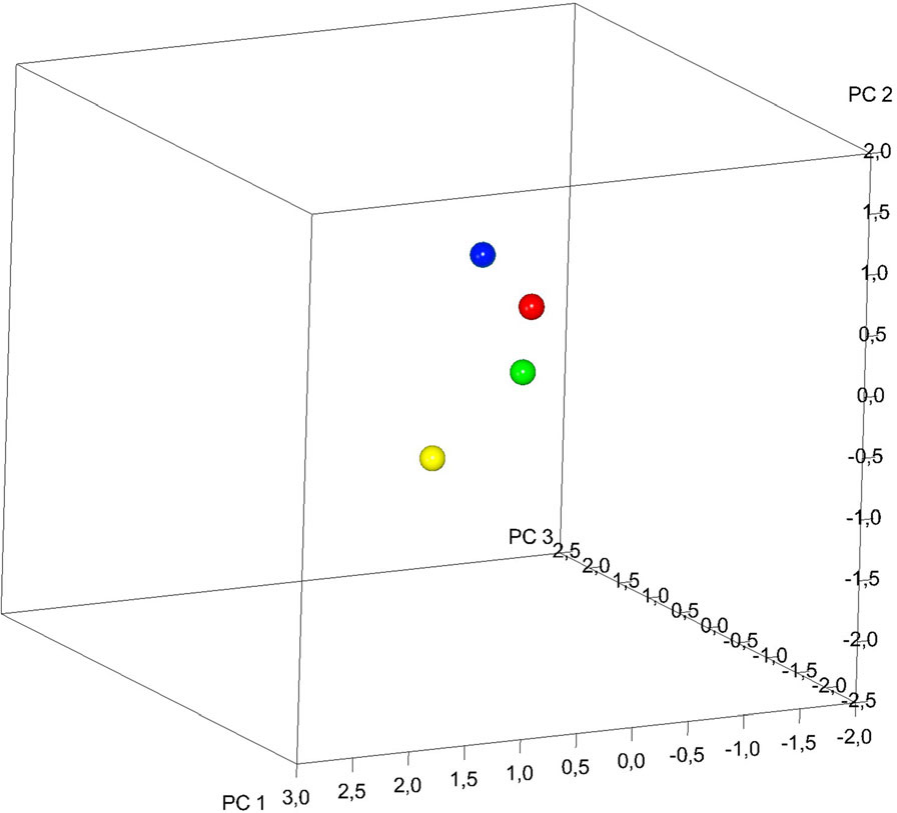
Plot distribution in the form-shape space of the average of the 4 subgroups (MG1 blue sphere, MG2 yellow sphere, CG2 green sphere, CG1 red sphere)

From the other two comparisons (MG1 vs CG1; 10,000 permutations; *P* = 0,003); and (MG2 vs CG2; 10,000 permutations; *P* = 0,007), significant differences in shape were shown. Both Marfan subgroups showed important reductions in the transversal plane associated with a deep palatal vault when compared to no syndromic individuals (Figs. 6a-c, 7a-c). While comparing the two control subgroups (CG1 vs CG2; 10,000 permutations; *P* = 0,073) no statistically significant differences in palatal shape are evidenced (Fig. 8a-c). Figure 9 represents the plot distribution in the form-shape space of the average of the 4 subgroups (MG1 blue sphere, MG2 yellow sphere, CG2 green sphere, CG1 red sphere).

## Discussion

The aim of the present study was to analyze maxillary morphology in 31 growing subjects with Marfan syndrome by means of 3D geometric morphometric evaluation of digital dental casts. Moreover our study highlighted the differences in Marfan patients according to two different stages of dentition (mixed and permanent dentition) by a comparison with a control group of no syndromic subjects.

In literature only one preliminary study by Laganà et al. [10] is reported about this topic. However this study analyzed only five growing MS subjects and compared them with a control group. They found a marked constriction of maxillary arch in Marfan group, and moreover a higher palatal vault. Except for this study, no data are reported in literature about 3D evaluation of palatal morphology in growing Marfan subjects. Several studies identified orofacial manifestations of MS and a general consensus has been reached in finding a significant correlation between MS and palatal shape alteration. Cephalometric parameters indicated a prevalence of a high and deep palate in 50% of subjects [20, 21]. A previous study by Cistulli et al. [22] assessed maxillary morphology of thirteen MS subjects, with a mean age of 32.3 years, using study casts and linear measurements like intercuspid distance, interpremolar distance, intermolar distance and maximum height of hard palate. They found a marked constriction of maxillary arch; by contrast a significant difference in the height of hard palate was not detected in comparison with a control group. However, the limitation of the study was that the evaluation of maxillary morphology used dental index in adult subjects usually affected by dental crowding and abnormality of tooth surfaces. Docimo et al. [9] actually found that Marfan patients have an important constriction of the maxilla associated with high palatal vault and some other typical clinical features. Nevertheless they evaluated thirty-two pediatric subjects (mean age 10.5 years), only based on a clinical evaluation of ogival palate and presence of cross-bite.

Still too few data in literature use a three-dimensional approach to study the characteristics of the upper jaw of growing patients and even less in syndromic patients [7].

**Fig. 10:**
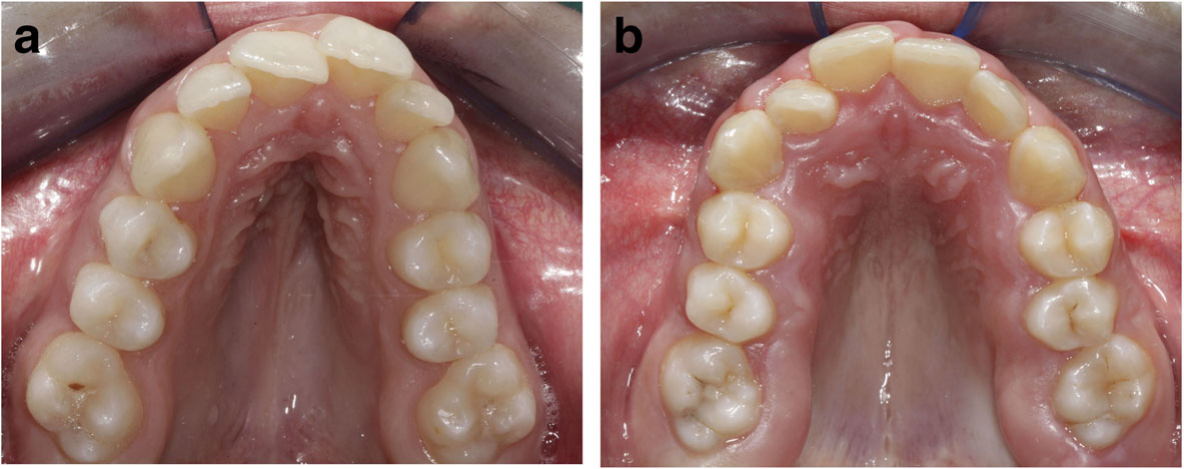
**a**-**b** Intraoral photographs of Marfan palates

To our knowledge this is the first study that used a 3D approach to evaluate the characteristics of patients with Marfan syndrome by comparing two distinct groups in different stages of dentition. This study revealed that the constriction of the maxilla as well as the depth of the palate is greater in the Marfan group in permanent dentition (Fig. 10a-b), rather than in Marfan patients with mixed dentition.

The precise mechanisms accounting for the worsening of palatal height and width from mixed to permanent dentition in patients with Marfan syndrome are not certain. It could be associated to their growth pattern or to a sutural disorder of ossification. Moreover numerous studies have suggested an important role of breathing disorders in the development craniofacial abnormalities, especially among Marfan patients [23].

Marfan patients showed an increased upper airway collapsibility during sleep related to the known connective tissue defect of the syndrome. Furthermore, high nasal airway resistance has been reported in Marfan syndrome and this appears to be mediated by maxillary constriction and high arched palate that is associated with the syndrome [24].

Data from the current study indicated that subjects with Marfan syndrome, when compared to healthy subjects, showed a typical palatal morphology. In particular, as shown in Figs. 6a-c, 7a-c, the morphological differences are localized on the transversal and the vertical planes. MG has a narrower and higher palatal vault compared with CG both in mixed dentition and in permanent dentition. Moreover, our study revealed that the most notable size contraction is localized especially in the posterior palatal region in MG.

Since the rarity of the syndrome, limitation of this study was the lack of longitudinally evaluation of the same group of Marfan patients. The observed dentition differences should, therefore, be interpreted with caution since they could reflect random differences due to sample selection.

## Conclusion

- Marfan subjects have a specific palatal morphology with several alterations in all three-dimension of the space when compared to healthy subjects.
- The constriction and the depth of the palatal vault in Marfan syndrome worsen from mixed dentition to permanent dentition more than in the control group of no syndromic subjects.
- Further studies are necessary in order to deeply understand maxillary growing pattern in Marfan subjects.
